# Tropics-wide intraseasonal oscillations

**DOI:** 10.1073/pnas.2511549122

**Published:** 2025-11-24

**Authors:** Jiawei Bao, Sandrine Bony, Daisuke Takasuka, Caroline Muller

**Affiliations:** ^a^Institute of Science and Technology Austria, Klosterneuburg 3400, Austria; ^b^Laboratoire de Météorologie Dynamique/Institut Pierre-Simon Laplace, Sorbonne University, CNRS, Paris 75252, France; ^c^Department of Geophysics, Graduate School of Science, Tohoku University, Sendai 980-8578, Japan

**Keywords:** tropical mean oscillations, radiative-convective equilibrium, Madden–Julian oscillation, intraseasonal oscillation

## Abstract

The tropical climate variability is characterized by various oscillations across a range of timescales. Oscillations that imprint the tropical mean state are generally attributed to slow processes, such as the seasonal cycle or interannual variability. Here, we identify a pronounced tropics-wide intraseasonal oscillation (TWISO) in satellite observations and reanalyses. This oscillation, with a period of 30 to 60 d, is evident across multiple variables and involves interactions between convection, radiation, surface fluxes, and large-scale circulation. It is primarily manifested as convective perturbations in the tropical Indo-Pacific warm pool accompanied by oscillations in the large-scale tropical overturning circulation. Here, we examine the relationship between TWISO, the Madden–Julian Oscillation (MJO), and the instability of radiative-convective equilibrium. Certain phases of TWISO coincide with specific phases of the MJO, suggesting a potential connection between the two. However, although the MJO can amplify the oscillation amplitude of TWISO, it is not essential for TWISO to occur. Finally, due to its broad manifestation across the tropics, TWISO potentially exerts widespread influence on tropical weather and climate at regional scales.

## Significance

Tropical climate variability is shaped by a range of oscillations, but most known modes influencing the tropical mean state occur on seasonal or interannual timescales. Here, we identify a pronounced tropics-wide intraseasonal oscillation (TWISO) with a 30 to 60-days period, evident in satellite observations and reanalysis data. TWISO emerges across multiple variables and reflects dynamic interactions among convection, radiation, and large-scale circulation. Its widespread expression suggests substantial impacts on tropical weather and climate at regional scales.

The tropical climate system is governed by two dominant mean overturning circulations: the Hadley circulation and the Walker circulation. These large-scale circulations are generally considered stable and evolve on relatively slow timescales (1, 2). Superimposed on this mean state, however, are various forms of regional climate variability occurring over fast timescales mainly driven by convectively coupled equatorial waves (3–6). Among these, the Madden–Julian Oscillation (MJO) stands out as the leading mode of intraseasonal variability in the tropics (4, 7). The MJO influences tropical weather and climate primarily through its eastward-propagating convective envelopes, which are coupled with large-scale atmospheric wave responses and drive teleconnection patterns across the tropics and beyond (5). Despite its critical role in modulating regional weather patterns, previous studies suggest that the MJO does not leave a significant imprint on the tropical mean (6). Instead, well-known large-scale variations in tropical-mean signals are typically attributed to slower processes, such as the seasonal cycle or the El Niño-Southern Oscillation. However, Bony et al. (8) recently detected pronounced intraseasonal variability in convective organization, with discernable radiative imprints at the tropical-mean scale. This raises the question of what type of intraseasonal phenomenon might be associated with this variability.

The thermodynamic structure of the tropical atmosphere results from a fundamental balance between latent heating from convection and cooling by radiation (9). Idealized simulations in radiative-convective equilibrium (RCE) reveal that the RCE state can be unstationary and exhibit oscillatory behavior (10). This phenomenon was first identified by Hu and Randall (10) who discovered pronounced 30 to 60-d oscillations in a single-column RCE model. In three-dimensional nonrotating RCE simulations, convection self-aggregates despite the homogeneous and stationary forcing (11–14). Emanuel et al. (15) hypothesized that self-aggregation is the manifestation of the linear instability of the RCE state. This is supported by several studies showing that convective self-aggregation oscillates and internal variability arises in simulations using a three-dimensional nonrotating RCE model coupled to a slab ocean (16, 17). Given the simplicity of the RCE framework, the instability is likely driven by fundamental interactions among radiation, convection, and surface fluxes. These idealized simulations therefore provide theoretical evidence supporting the existence of tropics-wide oscillations at intraseasonal timescales.

Regional synoptic variability is strongly influenced by convectively coupled equatorial waves (18, 19). These tropical waves are partially explained by the classical dry wave theory based on the shallow water equations (3, 20). However, certain phenomena, in particular the MJO, are not predicted by this theory (7, 21). To date, there is still no consensus on the driving mechanisms of the MJO (22, 23). It has been hypothesized that the MJO may be linked to the aforementioned low-frequency oscillations in RCE (10, 24). Further evidence supporting this idea comes from findings that convective self-aggregation, which originates in nonrotating RCE models as a nonpropagating phenomenon, can transform into MJO-like eastward-moving disturbances when Earth-like rotation is introduced in the same RCE models (25, 26). These findings raise the key question of whether observations indeed support tropics-wide intraseasonal oscillations, and, if so, how they connect to tropical waves and the instability of RCE.

In this study, we address these questions by using observations and reanalysis data. First, we identify the clear presence of tropics-wide oscillations at intraseasonal timescales in observations. We then show that this oscillation involves interactions between moist convection and the large-scale overturning circulation. Next, we explore the link between TWISO and the MJO, as well as its connections to the instability of the RCE system. Finally, we discuss the climate implications of TWISO.

## Observations Show Pronounced Signals of Tropics-Wide Intraseasonal Oscillations

**Fig. 1.**
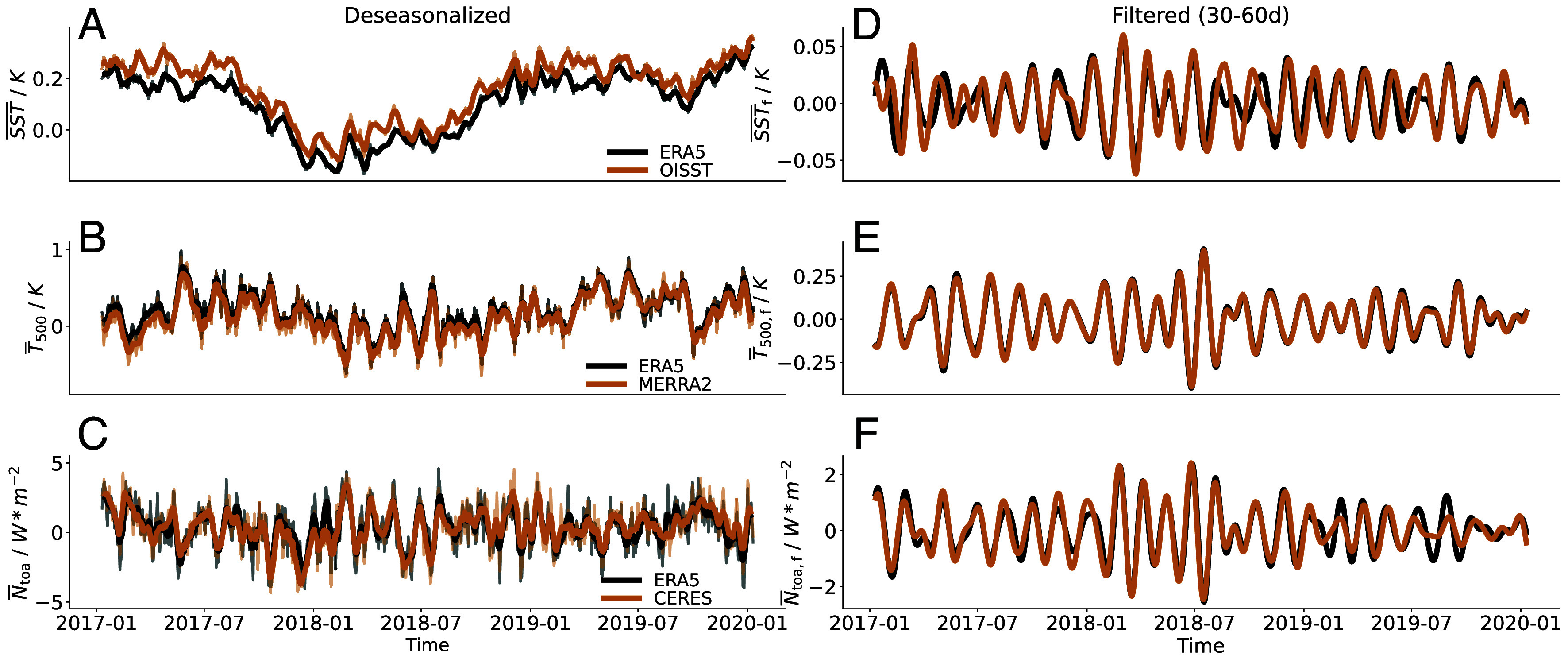
(*A*–*C*): Daily time series of the deseasonalized tropical mean sea surface temperature ($\bar{\mathrm{SST}}$), 500 hPa temperature (${\bar{T}}_{500}$), net top-of-atmosphere radiation (${\bar{N}}_{\text{toa}}$) averaged over the tropics (30^°^S to 30^°^N) from ERA5 and another source of dataset ($\bar{\mathrm{SST}}$ from OISST, ${\bar{T}}_{500}$ from MERRA2, ${\bar{N}}_{\text{toa}}$ from CERES). The deseasonalization is done by subtracting the daily climatological values over the 2001–2020 period. Thin lines: the original time series after deseasonalization; thick lines: the smoothed time series obtained by applying a 10-d running mean. (*D*–*F*): The same time series but filtered over the frequency range of 30 to 60 d by applying bandpass filtering with Fourier transform. For visualization purposes, the time series are shown only for the years 2017–2019. But the oscillations are robust across other years as well.

**Fig. 2.**
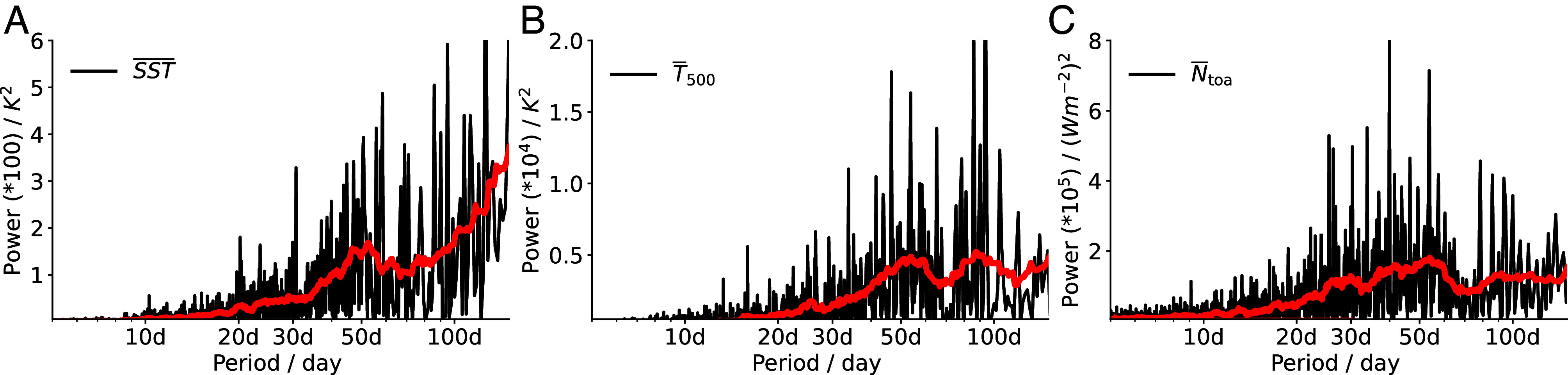
Power spectra of daily tropical-mean $\bar{\mathrm{SST}}$ (*A*), ${\bar{T}}_{500}$ (*B*), and ${\bar{N}}_{\text{toa}}$ (*C*) from ERA5, averaged over 30^°^S–30^°^N for 2001–2020. The data are deseasonalized by subtracting the 20-y daily climatological values. Red lines denote the 30-point smoothed periodogram.

We begin by analyzing the daily time series of the deseasonalized tropical-mean sea surface temperature ($\bar{\mathrm{SST}}$), air temperature at 500 hPa (${\bar{T}}_{500}$), and net top-of-atmosphere (TOA) radiation (${\bar{N}}_{\text{toa}}$) averaged over 30^°^N–30^°^S, using different satellite and reanalysis datasets. The deseasonalization is done by subtracting the daily climatological values over the 2001–2020 period. Contrary to our initial expectations, the tropical-mean time series of all three variables are not smooth, but instead, exhibit relatively fast oscillations (Fig. 1*A*–*C*). Power spectra of these daily time series reveals pronounced spectral peaks at intraseasonal timescales, particularly in the frequency range of 30 to 60 d, across all three variables (Fig. 2). Similar intraseasonal power peaks in ${\bar{N}}_{\text{toa}}$ are also reported by Bony et al. (8). To isolate this intraseasonal signal, we apply bandpass filtering with Fourier transform, retaining only the 30 to 60-d components (Fig. 1*D*–*F*). The resulting filtered time series, denoted with a subscript f, show that intraseasonal oscillations are key contributors to the original variability, accounting for 31% of the variance in ${\bar{N}}_{\text{toa}}$ and 26% in ${\bar{T}}_{500}$. In contrast, only 3% of $\bar{\mathrm{SST}}$ variance falls within this band, which is expected, as SST is largely dominated by interannual variability. In the following analyses, we focus specifically on oscillations within the intraseasonal timescale of 30 to 60 d. The above findings are robust across different observational and reanalysis datasets (Fig. 1 and *SI Appendix*, Fig. S1), so we will primarily use ERA5 data for the remainder of the study.

Given the pronounced intraseasonal oscillations observed in multiple tropical mean variables, we refer to them as Tropics-Wide IntraSeasonal Oscillations (TWISO). Remarkably, the oscillations are evident even when the variables are averaged as tropical mean values, highlighting a system-wide response across the tropics. This suggests that large-scale tropical circulations might oscillate as well. To investigate whether TWISO is associated with fluctuations of the tropical large-scale circulation, we compute two metrics that quantify the strength of the large-scale circulation: the pressure vertical velocity difference at 500 hPa, calculated as the tropical mean subsidence minus the mean ascent ($I={\bar{\omega }}_{\mathrm{dn}}-{\bar{\omega }}_{\mathrm{up}}$) (27), and the tropical mean surface wind speed ($\bar{V}$). These two circulation metrics capture different dynamical aspects. I is a measure of the strength of the tropical overturning circulations, including both the Hadley and Walker cells (27), whereas $\bar{V}$ primarily reflects the mean intensity of the trade winds. Both circulation metrics exhibit intraseasonal oscillations and are well correlated (*SI Appendix*, Fig. S2). The peak correlation (R≈0.66) of their filtered time series occurs with a temporal lag, with $I_{\text{f}}$ leading ${\bar{V}}_{\text{f}}$ by approximately 4 d. Thus, the above analysis confirms the presence of large-scale circulation oscillations. In the subsequent analysis, we use I as a proxy for the large-scale overturning circulation.

Next, we examine the relationships among these oscillating variables, focusing on the lead-lag correlation coefficients between the filtered large-scale overturning circulation ($I_{\text{f}}$) and the filtered tropical-mean variables (*SI Appendix*, Fig. S3*A*). Over periods of 30 to 60 d, they exhibit strong covariance. The strongest positive correlation (R≈0.69) between $I_{\text{f}}$ and ${\bar{T}}_{500,\text{f}}$ occurs when $I_{\text{f}}$ leads by 8 d, suggesting that widespread free-tropospheric warming follows the strengthening of the large-scale circulation. In contrast, the correlations between $I_{\text{f}}$ and ${\bar{\mathrm{SST}}}_{\text{f}}$ are generally opposite to those between $I_{\text{f}}$ and ${\bar{T}}_{500,\text{f}}$: the strongest negative correlation (R≈−0.51) occurs when $I_{\text{f}}$ leads ${\bar{\mathrm{SST}}}_{\text{f}}$ by 10 d. This suggests that, on intraseasonal timescales, SST responds to surface wind variations: Stronger large-scale circulation increases surface winds, which cools the SST by enhancing heat fluxes from the ocean to the atmosphere. Furthermore, the TOA radiation (${\bar{N}}_{\text{toa},\text{f}}$) decreases following the strengthening of large-scale circulation (R≈−0.66). Bony et al. (8) found that variability in the tropical mean TOA radiation budget is sensitive to the spatial organization of deep convection, which is itself likely influenced by the large-scale overturning circulation. Here, we confirm that convective organization, measured by the clustering index $I_{\text{org}}$, also oscillates at intraseasonal timescales, and is positively correlated with the large-scale circulation with a lag of approximately 10 d. It is in phase with TOA radiation budget and free-tropospheric temperature. This suggests that as the large-scale circulation strengthens, mesoscale convective organization increases, potentially contributing to the warming and drying of the free troposphere and leading to enhanced outgoing longwave radiation. The basic relationships also hold for the original data without filtering (*SI Appendix*, Fig. S3*B*). The cross-correlations still maximize at nearly the same lag and decay over intraseasonal timescales, confirming that these variables covary coherently on intraseasonal timescales.

Given the strong covariance among the tropical mean fields of these variables, we next focus on the spatial characteristics of the oscillations and investigate whether distinct spatial patterns emerge and dominate the tropical mean oscillation signal. To achieve this, we select $I_{\text{f}}$ as the common reference variable and regress the time series of $I_{\text{f}}$ onto the time series of other variables at each grid point across the entire tropical domain. Regions that significantly contribute to the mean oscillation signal are expected to stand out in the regression analysis. Using $I_{\text{f}}$ as the reference variable offers the advantage of directly representing the large-scale overturning circulation, which is inherently a spatially averaged concept. This simplifies the interpretation of the results. Furthermore, to identify the origin of the signal, we perform a lead-lag regression analysis spanning 12 d before to 12 d after day 0, where day 0 corresponds to the point with no lag between the local time series and the tropical mean time series. For clarity of interpretation, we describe the regression results in terms of the response of the regressed variable to an assumed peak in large-scale circulation at day 0. That is, we interpret day 0 as the time when the large-scale circulation reaches its maximum. Under this assumption, a positive regression coefficient indicates that the regressed variable (e.g. $T_{500,\text{f}}$) tends to exhibit a positive anomaly, while a negative coefficient indicates a negative anomaly.

**Fig. 3.**
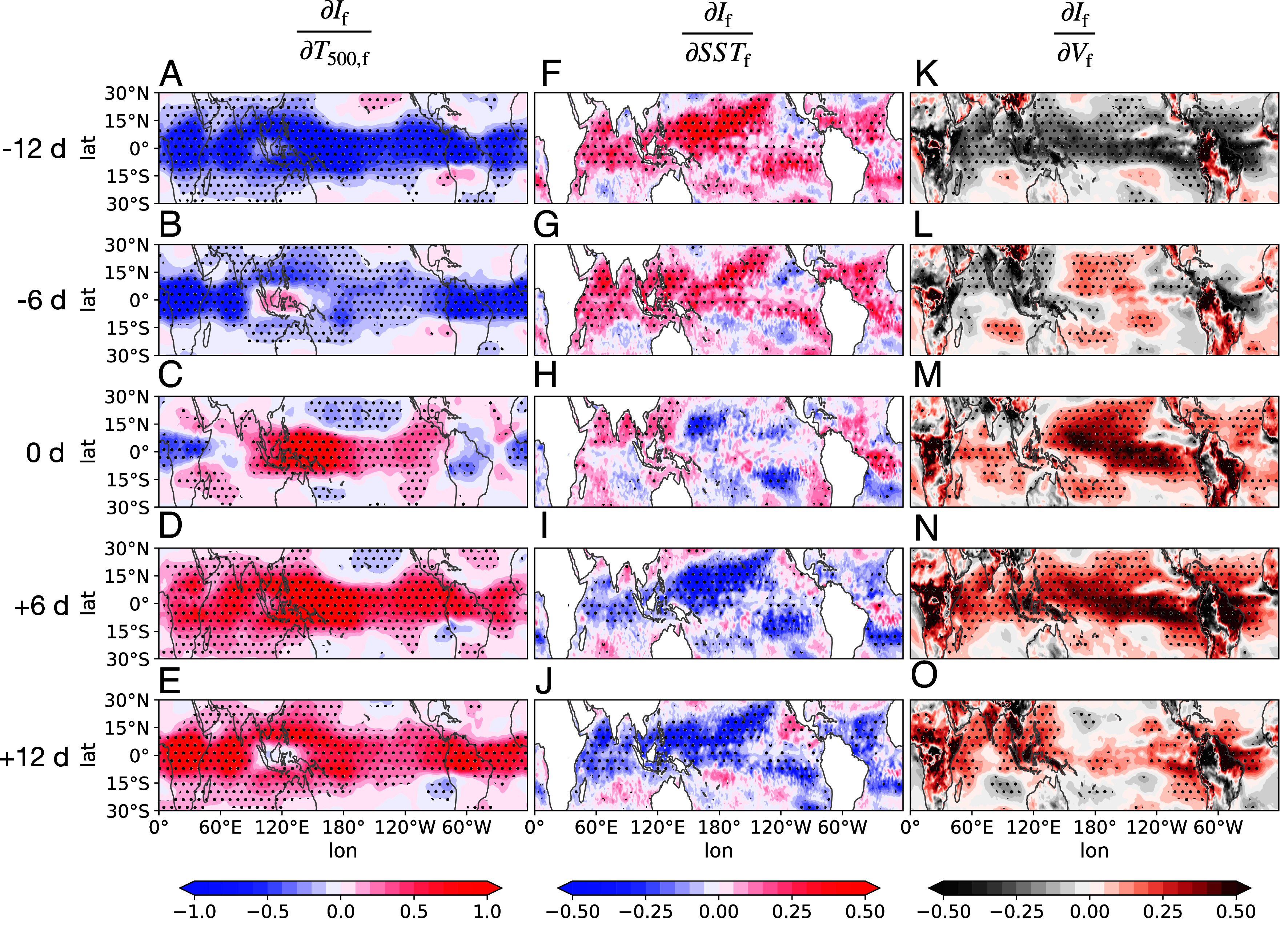
Spatial map of lead-lag regression coefficients by regressing the time series of filtered tropical mean overturning circulation index $I_{\text{f}}$ onto the time series of other variables, including 500hPa temperature: $T_{500,\text{f}}$ (*A*–*E*), sea surface temperature: $SST_{\text{f}}$ (*F*–*J*), and surface wind speed: $V_{\text{f}}$ (*K*–*O*) at each grid point across the entire tropical domain. All variables are filtered over 30 to 60 d. From *Top* to *Bottom* shows 12-d lag, 6-d lag, no lag, 6-d lead, and 12-d lead of $I_{\text{f}}$. Regression coefficients are normalized by the ratio of the SDs of the two variables, calculated across all spatial and temporal dimensions, to make them unitless. Regions where results are significant at the 95% confidence level (P<0.05) are indicated by stippling.

**Fig. 4.**
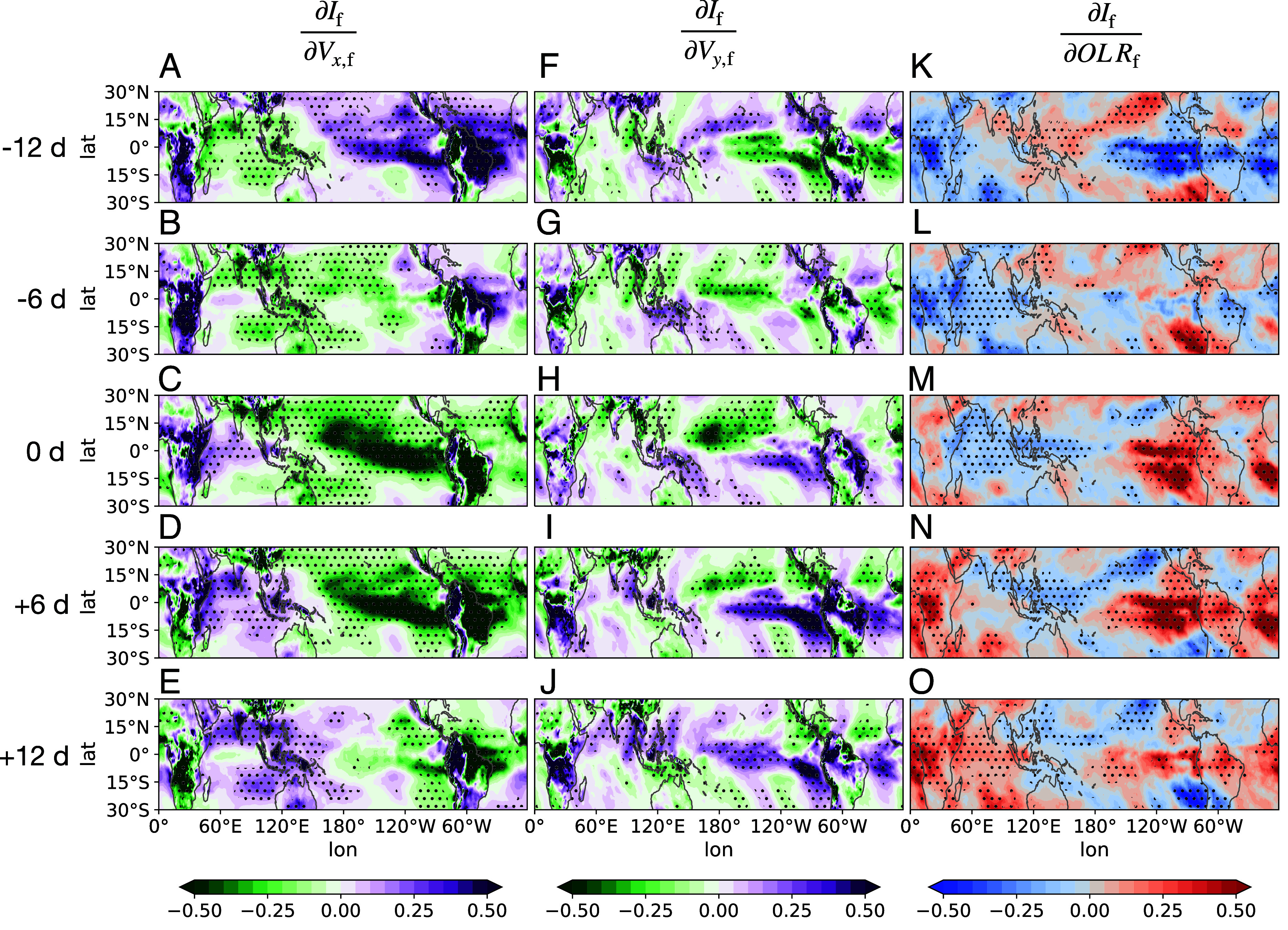
Spatial map of lead-lag regression coefficients by regressing the time series of filtered tropical mean overturning circulation index $I_{\text{f}}$ onto the time series of other variables, including surface zonal wind $V_{x,\text{f}}$ (*A*–*E*), surface meridional wind $V_{y,\text{f}}$ (*F*–*J*), and outgoing longwave radiation OLR_f_ (*K*–*O*) at each grid point across the entire tropical domain. All variables are filtered over 30 to 60 d. From *Top* to *Bottom* shows 12-d lag, 6-d lag, no lag, 6-d lead, and 12-d lead of $I_{\text{f}}$. Regression coefficients are normalized by the ratio of the SDs of the two variables, calculated across all spatial and temporal dimensions, to make them unitless. Regions where results are significant at the 95% confidence level (P<0.05) are indicated by stippling.

We first focus on the regression of $I_{\text{f}}$ onto $T_{500,\text{f}}$ ($\frac{\partial I_{\text{f}}}{\partial T_{500,\text{f}}}$). Approximately 12 d prior to the peak in large-scale circulation intensity (day 0), the atmosphere shows a widespread cold anomaly, coinciding with a broad-scale warm anomaly of tropical SSTs (Fig. 3 *A* and *F*). By day −6, an initial warm perturbation begins to emerge in the free troposphere above the Maritime Continent. This atmospheric warm perturbation further propagates spatially, extending across the tropical Pacific within 6 d and covering the entire tropics within 12 d. By day +6, positive regression coefficients are observed almost uniformly across the tropics. The initial warm perturbation over the Maritime Continent is associated with enhanced deep convection there. The following atmospheric temperature response reflects a classical Weak Temperature Gradient mechanism, where fast-moving gravity waves, triggered by deep convective perturbations, efficiently distribute warming from convective regions throughout the tropics (28, 29). Simultaneously, there is a widespread positive anomaly in surface wind speed at around day 0 (Fig. 3*M*), following the initial convection enhancement over the Maritime Continent. This strengthening of surface winds is further evident in the regression with near-surface zonal ($V_{x,\text{f}}$) and meridional ($V_{y,\text{f}}$) winds (Fig. 4*A*–*J*). Strong easterly anomalies (indicated by negative regression coefficients) appear over the central and eastern Pacific, while westerly anomalies (positive coefficients) dominate over the Indian Ocean. These patterns emerge and intensify around day 0, following the onset of convection over the Maritime Continent. The strong easterly anomalies over the Pacific, a region typically characterized by climatological easterly trade winds and the lower branch of the Walker circulation, suggest an amplification of large-scale zonal overturning. Similarly, regression analysis with meridional wind shows strengthened meridional flow, indicating an intensification of the Hadley circulation. Together, these results suggest that following the initial convection increase over the Maritime Continent, large-scale overturning circulations, including both Walker and Hadley circulations become more vigorous. Meanwhile, $SST_{\text{f}}$ oscillates out of phase with $T_{500,\text{f}}$ (Fig. 3*F*–*J*). Warm perturbation of $SST_{\text{f}}$ dominate at day −12. However, as $I_{\text{f}}$ intensifies, $SST_{\text{f}}$ transitions into its negative phase. This transition is closely associated with the intensification of large-scale circulation, which strengthens surface winds and subsequently cools the sea surface by evaporation.

## Links Between TWISO and MJO

The above regression results mainly highlight the stationary components of TWISO. In contrast, the regression with outgoing longwave radiation (OLR) ($\frac{\partial I_{\text{f}}}{\partial OLR_{\text{f}}}$) reveals a pronounced eastward propagation signal (Fig. 4*K*–*O*). On day −12, negative OLR perturbations, indicating enhanced convection, are primarily concentrated over the African continent. This enhanced convection propagates eastward, reaching the Maritime Continent by day 0 before continuing toward the Pacific Ocean in the subsequent days. The eastward propagation is further confirmed by the time evolution of OLR anomalies composited around the days of maximum tropical overturning circulation (*SI Appendix*, Fig. S4).

The eastward propagation of negative OLR perturbations is reminiscent of the MJO. Therefore, we examine the relationship between TWISO and the MJO. The MJO is characterized using the Real-time Multivariate (RMM) Index (30), derived from the first two principal components of OLR and zonal winds at 850 hPa and 200 hPa. RMM1 and RMM2 together describe the dominant variability of the MJO, capturing its characteristic eastward propagation: Positive RMM1 corresponds to enhanced convection over the Indian Ocean and the Maritime Continent; Positive RMM2 corresponds to enhanced convection over the western Pacific. To assess the connection between the MJO and TWISO, we compute lead-lag correlations between the oscillations and RMM1/RMM2 (*SI Appendix*, Fig. S5 *A*–*B*). The results reveal significant correlations: RMM1 leads TWISO, showing a positive correlation with ${\bar{T}}_{500,\text{f}}$, but a negative correlation with ${\bar{\mathrm{SST}}}_{\text{f}}$ and ${\bar{N}}_{\text{toa},\text{f}}$. The peak absolute correlations reach 0.6 for all three variables. RMM2 exhibits similar correlations but lags significantly behind TWISO. Because the RMM index is dominated by its large-scale circulation component (31, 32), we repeat the lead-lag correlation analysis using the purely OLR-based MJO index (OMI) in *SI Appendix*, Fig. S5 *C* and *D*. RMM and OMI share the same phase-space framework and therefore are directly comparable: OMI2 is analogous to RMM1 and −OMI1 is analogous to RMM2 (32). The relationships between TWISO and OMI exhibit the same lag structure as TWISO and RMM, with peak absolute values slightly reduced to 0.5. In addition to the correlation analysis, the occurrence of the MJO is further confirmed by regressing $I_{\text{f}}$ onto the MJO-filtered surface zonal ($V_{x,\text{mjo}}$) and meridional winds ($V_{y,\text{mjo}}$), both of which exhibit a strong eastward propagation signal following active MJO convection (*SI Appendix*, Fig. S6).

How are the TWISO and the MJO related? One hypothesis is that TWISO is driven by the nonlinear interaction between the MJO and the large-scale overturning circulation. Fundamentally, the interaction between a propagating wavenumber-1 disturbance (e.g. the MJO) and a stationary wavenumber-1 feature (e.g. the Indo-Pacific warm pool and the associated Walker circulation) can generate a time-varying, wavenumber-zero response (e.g. the TWISO) (33). This nonlinear interaction can emerge from the coupling between convection and the large-scale circulation, potentially leading to a resonant amplification of intraseasonal variability and giving rise to TWISO. In the tropics, the Indo-Pacific warm pool sustains persistent convection throughout the year. The convection is associated with large-scale circulation and convergence into these regions. As the MJO propagates eastward across the Indian Ocean and the Maritime Continent, it further amplifies the convection in these regions and strengthens the large-scale circulation. Conversely, when the MJO moves away from the warm pool region, convective activity and the strength of the associated circulation both diminish. As it is in deep convective regions, particularly over the warm pool, that the free tropospheric temperature is the most strongly coupled to the boundary layer and ocean SST, convective perturbations in these regions exert a dominant influence on free-tropospheric temperature and large-scale circulation (34, 35). In contrast, convection in other regions is less effective due to the absence of this strong coupled dynamic (36).

## RCE Instability

**Fig. 5.**
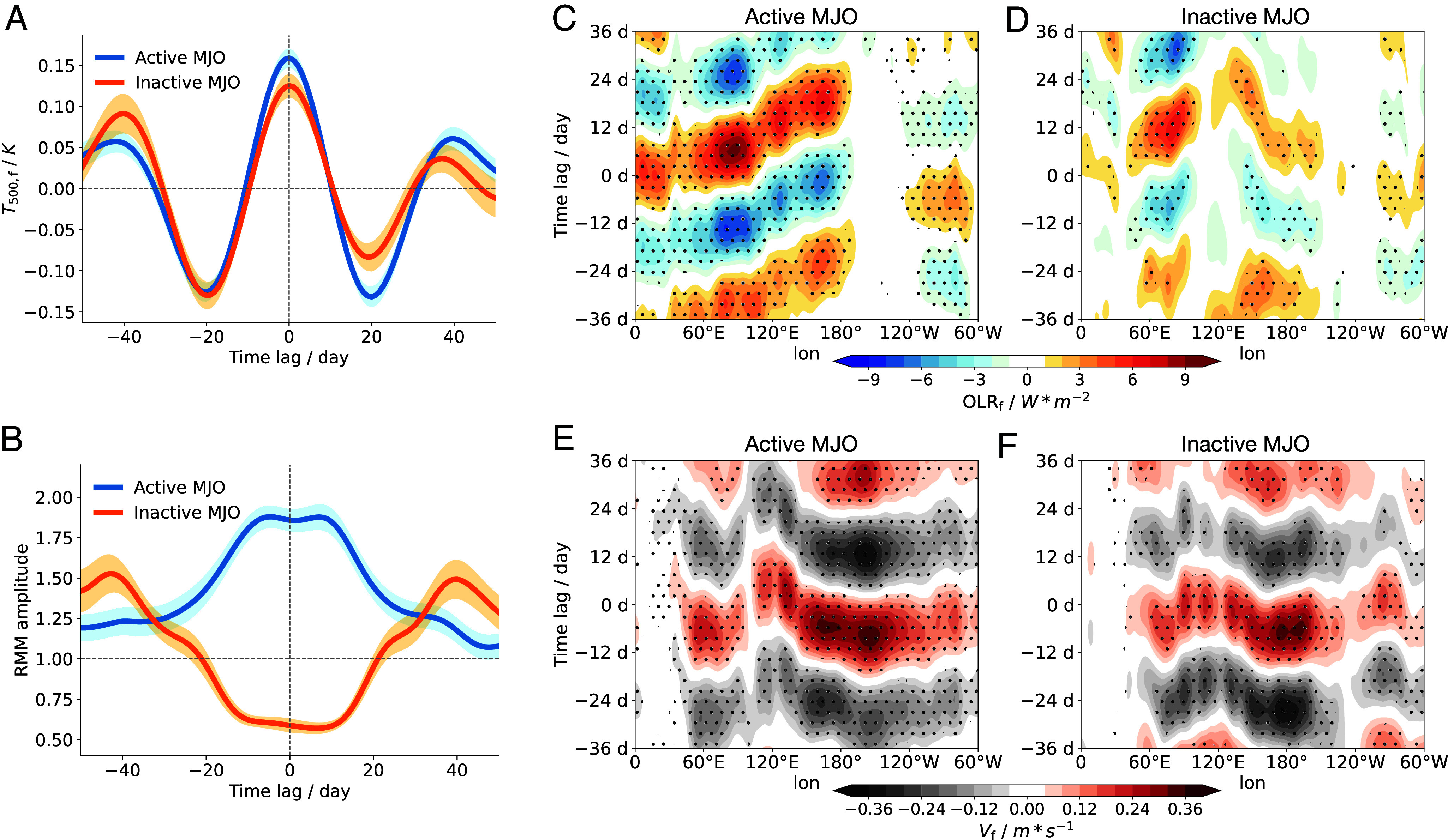
(*A* and *B*) Time series of ${\bar{T}}_{\text{500,f}}$ (*A*) and RMM amplitude (*B*) composited for active and inactive MJO periods. Day 0 corresponds to local peaks in ${\bar{T}}_{\text{500,f}}$, defined as days when ${\bar{T}}_{\text{500,f}}$ exceeds its values during both the preceding and following 10 d. An active (inactive) MJO event is defined as a case where the RMM amplitude, smoothed with a 10-d running mean, remains *Above* (*Below*) 1 throughout the 20-d window. Shadings show the SE of the mean across the composited events. (*C* and *D*) Hovmöller diagrams of composite OLR_f_ associated with ${\bar{T}}_{\text{500,f}}$ peaks at day 0 during active MJO (*C*) and inactive MJO (*D*) periods. OLR is averaged meridionally between 10^°^S and 10^°^N and filtered in the 30 to 60-d band. Stippling indicates regions where anomalies are statistically significant at the 95% confidence level (P<0.05). (*E* and *F*) Same as (*C* and *D*), but for filtered surface wind speed anomalies ($V_{\text{f}}$), averaged between 20^°^S and 20^°^N.

Could TWISO occur independently of the MJO? Yes. Fig. 5*A* shows the time series of $T_{\text{500,f}}$ composited for active and inactive MJO periods. Day 0 marks the peak of the $T_{\text{500,f}}$ oscillation cycle. An active MJO event is defined as one in which the smoothed RMM amplitude remains above 1 throughout the 20-d window centered on day 0, while an inactive event is one in which it remains below 1 over the same period. Under this definition, the mean RMM amplitude for inactive MJO cases remains below 1 across the entire 40-d window (from 20 d before to 20 d after day 0) (Fig. 5*B*). During active MJO periods, there is a clear eastward propagation of OLR anomalies from the Indian Ocean to the Pacific, consistent with the canonical MJO signal (Fig. 5*C*). In contrast, the eastward propagation is absent during inactive MJO periods (Fig. 5*D*). Interestingly, in addition to the stationary OLR oscillation over the Indo-Pacific warm pool, a westward-propagating OLR signal emerges, originating in the central and eastern Pacific and extending into the western Pacific. Similar intraseasonal westward-propagating patterns have been reported over the western Pacific in recent studies and are attributed to moisture modes and equatorial Rossby wave dynamics (18, 37, 38). While the exact mechanism requires further investigation, our results suggest that this westward-propagating pattern is associated with the moisture modes which is facilitated by TWISO, as intensification of large-scale circulation strengthens surface easterlies in the Pacific, which may reinforce the propagation through horizontal advection of moist static energy (MSE). Clear oscillations of surface wind speed across the tropics further confirm the presence of TWISO during both active and inactive MJO periods (Fig. 5 *E* and *F*). Surface flux anomalies also exhibit westward propagation (*SI Appendix*, Fig. S7), with the signal more pronounced during active MJO phases. Nevertheless, OLR anomalies maintain strong eastward propagation during active MJO. This contrast suggests that the oscillation during inactive periods may represent the baseline state, with the MJO superimposing additional complexity with an eastward-propagating component.

Although the oscillation amplitude of $T_{\text{500,f}}$ increases during active MJO periods, the intraseasonal variability persists even when the MJO is inactive (Fig. 5*A*). This suggests that while the MJO can modulate and amplify TWISO, its presence is not essential for TWISO to occur, as long as convective intensity continues to oscillate over the warm pool. In such cases, TWISO may be more analogous to the self-sustained intraseasonal oscillations seen in idealized simulations (10, 17, 39). Idealized modeling studies suggest that RCE is inherently unstable due to the nonlinear interactions between convection, surface fluxes, and radiation. This instability influences the mean state of the atmosphere across various timescales and affects the large-scale circulation (16, 40). To understand this, we focus on the domain-mean diabatic heating of the atmosphere (${\bar{Q}}_{\text{atm}}$) in RCE which can be expressed as the sum of the surface heat flux (${\bar{Q}}_{\text{sfx}}>0$), net radiative cooling of the atmosphere (${\bar{Q}}_{\text{rad}}<0$):

[1] $$\begin{array}{c} \begin{array}{c} {\bar{Q}}_{\text{atm}}={\bar{Q}}_{\text{sfx}}+{\bar{Q}}_{\text{rad}}. \end{array} \end{array}$$

**Fig. 6.**
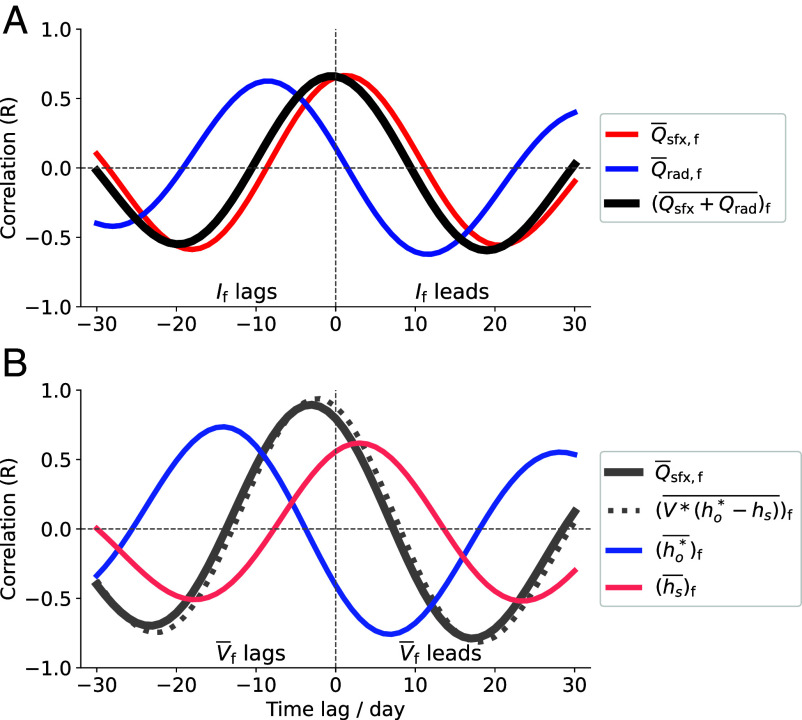
(*A*) Lead-lag correlation coefficient between the filtered tropical mean overturning circulation index $I_{\text{f}}$ and the filtered tropical mean surface fluxes ${\bar{Q}}_{\text{sfx},\text{f}}$, tropical mean net atmospheric radiation ${\bar{Q}}_{\text{rad},\text{f}}$ and the sum of the two ${\left(\bar{Q_{\text{sfx}}+Q_{\text{rad}}}\right)}_{\text{f}}$. (*B*) Lead-lag correlation coefficient between the filtered tropical mean surface wind speed ${\bar{V}}_{\text{f}}$ and ${\bar{Q}}_{\text{sfx},\text{f}}$, the approximations of surface fluxes $({\bar{V*(h_{o}^{*}-h_{s}))}}_{\text{f}}$, tropical mean saturation moist static energy of ocean surface ${\left(\bar{h_{o}^{*}}\right)}_{\text{f}}$ and moist static energy of the atmosphere at 2 m ${\left(\bar{h_{s}}\right)}_{\text{f}}$. All variables are filtered over 30 to 60 d.

In RCE, the time-mean ${\bar{Q}}_{\text{atm}}=0$ at steady state. However, because convection, radiation, and surface fluxes operate on different timescales, their temporal mismatch can produce transient energy imbalances in the atmospheric column. These imbalances can drive changes in the large-scale circulation. Indeed, we find that $I_{\text{f}}$ is well correlated and in phase with the total diabatic heating from surface fluxes and radiation ${\left(\bar{Q_{\text{sfx}}+Q_{\text{rad}}}\right)}_{\text{f}}$, which is itself closely aligned with the surface flux component ${\bar{Q}}_{\text{sfx,f}}$ (Fig. 6*A*). This suggests that intraseasonal variations in large-scale circulation are primarily driven by changes in surface fluxes, which, over longer timescales, are balanced by atmospheric radiative cooling (41). Although horizontal energy transport to the extratropics contributes to the time-mean tropical energy budget, it is a sink term that responds to the energy imbalance and is much smaller than surface fluxes and radiation, especially on intraseasonal timescales (42, 43).

The above mechanism highlights the importance of internal feedbacks, in which surface fluxes are crucial in facilitating TWISO. Then, the question is what drives the changes in surface fluxes at intraseasonal timescales. Changes in surface fluxes are often attributed to variations in surface wind speed. However, our results show that perturbations in surface fluxes actually precede those in surface wind speed (*SI Appendix*, Fig. S8). To understand the relationship, we consider the surface flux approximation equation:

[2] $$\begin{array}{c} \begin{array}{c} Q_{\text{sfx}}=C_{D}*V*(h_{o}^{*}-h_{s}), \end{array} \end{array}$$

where $Q_{\text{sfx}}$ is approximated by V times air–sea enthalpy differences ($h_{o}^{*}-$$h_{a}$), $C_{D}$ is a dimensionless coefficient, $h_{s}$ is the MSE near the surface and $h_{o}^{*}$ is the saturation MSE at the ocean surface. Based on Eq. **2**, V has two opposing effect on $Q_{\text{sfx}}$: While enhanced V tends to increase $Q_{\text{sfx}}$ (a positive feedback), strong V can also weaken $Q_{\text{sfx}}$ by reducing $h_{o}^{*}-h_{a}$ (a negative feedback) as it increases $h_{a}$ while decreasing $h_{o}^{*}$. These opposing effects suggest that, eventually, despite strong winds, surface fluxes will begin to decrease as the air–sea enthalpy differences are diminished by intense winds. The above relationships are evident when these variables are averaged at the tropical mean scale (Fig. 6*B*). These opposing feedbacks constitute a potential mechanism for surface flux oscillations, which can facilitate the oscillations of the entire system. *SI Appendix*, Fig. S8 shows that regions where changes in surface fluxes precede changes in surface winds are primarily located over the eastern Pacific Ocean and tropical land areas, including the Maritime Continent, Southeast Asia, and India. Over the oceans, surface flux changes initiate in the eastern Pacific around day −12 and progressively expand westward toward the western Pacific over the following 5 to 10 d. This westward progression is evident in the surface flux Hovmöller diagrams for both active and inactive MJO cases (*SI Appendix*, Fig. S7), suggesting that it is a robust feature arising from the interaction among surface fluxes, surface wind speed, and air–sea enthalpy differences.

In this framework, internal feedbacks between convection, radiation, and surface fluxes are central to driving the TWISO. Their interactions generate temporal imbalances in atmospheric energy, which in turn modulate the strength of the large-scale overturning circulation. Convection and circulation respond rapidly to perturbations in surface fluxes, triggering a broader, tropics-wide response involving atmospheric temperature, SST, and radiation. Surface fluxes mediate the energy exchange between the ocean and atmosphere. Increased surface fluxes initially enhance convection and circulation, but simultaneously reduce the air–sea enthalpy difference. This forms a negative feedback loop that ultimately weakens the surface fluxes and reverses the cycle.

Both the MJO-related and RCE-instability mechanisms converge on the idea that TWISO arises from the coupling between convection over the warm pool and the large-scale tropical overturning circulation. The key difference lies in what initiates the convective oscillation over the warm pool. The MJO-related mechanism highlights the nonlinear interaction between the MJO and the large-scale overturning circulation, which can stem from fundamental principles of wave dynamics. However, our results indicate that the presence of MJO is not necessary for TWISO to occur. Additionally, intraseasonal oscillations have been identified in idealized simulations without rotation, and thus without the MJO (10, 16, 17, 39). This supports the idea that such oscillations can arise from internal feedbacks alone, consistent with the RCE-instability mechanism. While this evidence favors RCE instability as a plausible driver of TWISO, the exact role of the MJO remains uncertain. Observational data alone may not be sufficient to disentangle these contributions. Therefore, we leave this as an open question, and recommend that future work combine modeling and mechanism-denial experiments to clarify the relative roles of the MJO, RCE instability, and their possible interactions.

## Schematic of TWISO

**Fig. 7.**
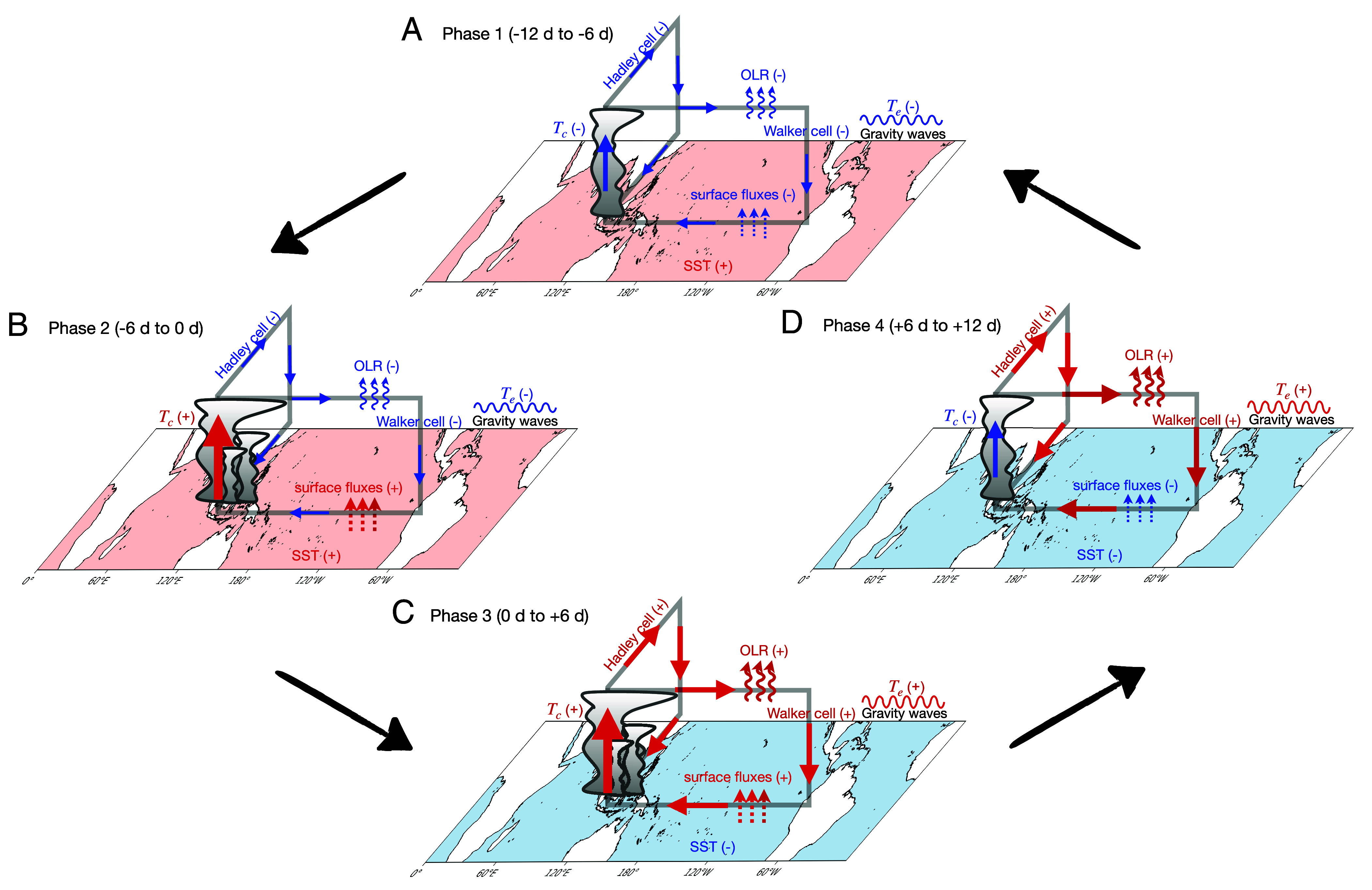
Schematic of TWISO. (*A*-*D*) describe the tropical climate response during the four phases of TWISO. Red (blue) colors indicate that the corresponding processes have positive (negative) perturbations. $T_{c}$ and $T_{e}$ represent atmospheric temperature near convection and the environment temperature respectively. Note that MJO is not shown in this schematic because it is not a necessary condition for TWISO to occur. However, when MJO is active, TWISO occurs with enhanced amplitude.

The key processes involved in TWISO are illustrated in Fig. 7 in four phases. We begin with phase 1, characterized by suppressed convection over the Indo-Pacific warm pool and a weak large-scale circulation (Fig. 7*A*). Atmospheric stability is low due to a cool atmosphere over warm SST. This thermodynamic configuration enhances air–sea enthalpy differences, subsequently driving a shift in surface flux perturbations and resulting in increased surface fluxes during phase 2, which occurs before a significant large-scale surface wind response (Fig. 7*B*). During phase 1, convection is less organized and OLR is reduced. Then in phase 2 convection in the warm pool intensifies, leading to a positive temperature anomaly in the free troposphere in the vicinity of convection. This warming anomaly is communicated across the tropics via fast-propagating gravity waves, producing tropics-wide atmospheric warming within approximately 10 d in phase 3 (Fig. 7*C*). Concurrently, large-scale circulations, including both the Hadley and Walker circulations, strengthen. These enhanced circulations amplify subsidence over nonconvective regions, promoting drying. The combined effects of warming and drying decrease relative humidity and increase OLR. These changes are further reinforced by an increase in mesoscale convective organization during this phase. At the surface, stronger winds associated with the intensified large-scale circulation decrease the SST. The combination of a warmer atmosphere and a cooler ocean surface increases static stability and decreases air–sea enthalpy differences, contributing to the subsequent weakening of convection and large-scale circulation.

In phase 4, the atmosphere is more stable (Fig. 7*D*). Again, surface flux perturbations respond first and transition to their negative phase. Then convection is suppressed over the Maritime Continent, generating a negative temperature anomaly near the convection. This cooling anomaly propagates across the tropical free-troposphere. The reduced convection is associated with a weakening of large-scale circulation and subsidence in nonconvective regions, leading to moistening of the free troposphere and a decrease in OLR (back to phase 1). Meanwhile, diminished surface wind speeds allow SSTs to rise, which decreases static stability as the troposphere cools relative to the ocean surface. The increase in SST leads to a larger air–sea enthalpy difference. This, combined with the reduced stability, fosters a gradual reintensification of convection and the large-scale circulation, thereby completing the oscillation cycle.

The MJO often propagates eastward in tandem with the TWISO, although its presence is not necessary for TWISO to occur. When the MJO coincides with the TWISO, phase 1 is marked by the MJO convective envelope positioned over South America. Between phases 1 and 2, it propagates eastward from South America toward the Indian Ocean, with the eastern edge of its convective envelope already bordering the convective center over the Maritime Continent. In phase 3, the MJO continues its eastward progression from the Indian Ocean to the Maritime Continent. Finally, in phase 4, the MJO reaches the central and eastern Pacific before transitioning back to phase 1.

## Implications of TWISO

We identify a pronounced TWISO in satellite observations and reanalysis data. This oscillation has a period of 30 to 60 d and manifests itself in multiple variables involving convection, radiation, surface fluxes, SST, and large-scale circulation. It is primarily manifested as convective perturbations over the Indo-Pacific warm pool, along with associated oscillations in the large-scale tropical overturning circulation. We find that the MJO amplifies the oscillatory behavior of the TWISO, although its presence is not a necessary condition for TWISO to occur.

While the exact mechanism initiating TWISO remains challenging to pinpoint, the primary focus of this paper is the identification of TWISO as a stationary oscillatory component at intraseasonal timescales spanning the entire tropics. Traditionally, large-scale tropical mean responses have been attributed to slower modes of climate variability. The intraseasonal oscillations of the large-scale circulation as identified here challenge the conventional view of the Hadley cell as a stable system over shorter timescales (44, 45), highlighting the role of dynamical interactions between moist convection and the large-scale circulation.

TWISO is expected to have important implications for tropical weather and climate. First, oscillations in large-scale circulation can drive coordinated responses across the tropical climate system. This causes many processes to covary, which may enhance intraseasonal predictability. Second, strong oscillations may push the system far from its mean state, leading to significant departures from typical conditions and increasing the likelihood of extreme events. In addition, intraseasonal variability in tropical mean top-of-atmosphere radiation suggests that TWISO modulates the tropical energy balance. By modulating energy fluxes, TWISO may affect exchanges with the extratropics, potentially impacting global weather and climate patterns.

Finally, the identification of TWISO raises many questions. First, although idealized RCE simulation do predict some domain-mean internal variability driven by mechanisms similar to those of TWISO, it remains unknown whether climate models used in a realistic framework can reliably represent TWISO. Second, a separate line of research focuses on the intraseasonal variability of atmospheric angular momentum (AAM) (46–48), showing that the MJO accounts for only about half of the intraseasonal AAM variance, while extratropical mountain torques play a significant role. It would be interesting to investigate the interactions between the TWISO and the AMM, and whether extratropical processes also contribute to the dynamics of TWISO. Third, the driving mechanism and the relationship between TWISO and the MJO require further clarification. Our results suggest that TWISO is more fundamental than the MJO, because it can occur in the absence of MJO. However, it remains unclear whether the ability to simulate the MJO depends on the representation of TWISO, or vice versa. A deeper understanding of TWISO could offer new insights into the driving mechanism of MJO, which has been a long-standing challenge in tropical climate research (22, 23). Further research is crucial to uncover the underlying mechanisms of TWISO, its representation in models, and its broader impacts.

## Materials and Methods

### Data.

All analyses in this study are based on daily ERA5 reanalysis data from 2001 to 2020 (49), unless explicitly indicated otherwise. The key variables related to TWISO, namely SST, air temperature, surface energy fluxes, surface wind speed, and radiation budgets are in good agreement with other observational and reanalysis data (Fig. 1 and *SI Appendix*, Fig. S1). For example, radiation is validated onto satellite observations of CERES (50) and daily SST are compared with DOISST (51). Free-tropospheric thermodynamical variables are compared with MERRA2 reanalysis data (52). For visualization purposes, the time series in Fig. 1 and *SI Appendix*, Fig. S2 are shown only for the period 2017–2019. However, similar oscillations are evident in other years as well. All other figures in the manuscript are based on the full 20-y dataset.

### Analysis Method.

All data are deseasonalized by subtracting their 20-y daily climatology (2001–2020) prior to further analysis. Tropical means of each variable are computed as the area-weighted average over the latitude band of 30^°^S to 30^°^N. To isolate the intraseasonal timescale of 30 to 60 d, we first apply a bandpass filtering with Fourier transform to retain only the 30 to 60-d signals. The filtered variables are indicated with a subscript f. We then perform either correlation analysis or regression analysis, with lead-lag regression used to identify the spatial structures of variables that accompany or precede tropical mean oscillations.

Tropical mean overturning circulation is quantified as the difference in pressure vertical velocity at 500 hPa, between regions of mean subsidence and mean ascent, averaged over the latitude band 20^°^S–20^°^N: $I={\bar{\omega }}_{\mathrm{dn}}-{\bar{\omega }}_{\mathrm{up}}$ (27). We restrict the calculation to this narrower tropical band to avoid contamination from mid-latitude cyclones, which commonly affect the 20^°^ to 30^°^ latitude range and can significantly bias the estimate of I. For the regression analysis, we choose the filtered tropical mean overturning circulation ($I_{\text{f}}$) as the reference variable and regress the time series of $I_{\text{f}}$ onto the time series of other variables at each grid point across the entire tropical domain. Other oscillating variables, such as ${\bar{T}}_{500,\text{f}}$, could also serve as the reference variable provided they exhibit a clear oscillation signal in their tropical mean averages. Using $I_{\text{f}}$ as the reference variable offers the advantage of directly representing the large-scale circulation, which is inherently a spatially averaged concept. This simplifies the interpretation of the regression results. Since the regression involves variables with different physical units, we normalize the regression coefficients so that they are unitless. This is achieved by rescaling the coefficients using the ratio of the SDs of the two variables involved. For example, when regressing $I_{\text{f}}$ against ${\bar{T}}_{500,\text{f}}$, the resulting regression coefficients are multiplied by the ratio of the SD of ${\bar{T}}_{500,\text{f}}$ to that of $I_{\text{f}}$. The SDs are computed over all spatial and temporal dimensions, yielding a single normalization factor that is uniform across space. This normalization does not alter the spatial patterns of the regression coefficients.

As tropical-mean oscillations emerge from regional-scale variability, the regression coefficient quantifies the extent to which fluctuations at each specific location contribute to the overall variability of the tropical-mean oscillation. To identify the origin of the signal, we perform a lead-lag regression analysis spanning 12 d before to 12 d after day 0, where day 0 corresponds to the point with no lag between the local time series and the tropical mean time series. Regions where results are significant at the 95 % confidence level (P<0.05) are indicated by stippling. Because filtering introduces high autocorrelation, thereby reducing the effective sample size, we follow the method described in Livezey and Chen (53) to adjust the degrees of freedom used for *P*-value calculation by accounting for the autocorrelation and interdependence of each variable.

MJO is detected with two indices: the Real-time Multivariate (RMM) Index (30) and the OLR-based MJO index (OMI) (32). The RMM index locates the enhanced MJO convective envelope and quantifies its strength using an empirical orthogonal function (EOF) analysis. It is derived from the first two principal components (PC) of OLR and zonal winds at 850 hPa and 200 hPa from 15^°^S to 15^°^N. The pair of PC time series that form the index are called the Real-time Multivariate MJO series 1 (RMM1) and 2 (RMM2). The RMM amplitude, defined as (RMM1^2^+RMM2^2^)^1/2^, is a measure of the MJO intensity. As RMM index is dominated by the circulation component (31), we also include OMI in the analysis. The OMI is constructed in the same EOF phase-space framework but is a purely OLR-based MJO metric. Note that RMM and OMI are directly comparable: OMI2 is analogous to RMM1 and -OMI1 is analogous to RMM2 (32). The RMM index is available from the Bureau of Meteorology of Australia (https://www.bom.gov.au/climate/mjo/) and the OMI is available from NOAA website (https://psl.noaa.gov/mjo/mjoindex/). For the space-time regression analysis, the MJO signal is extracted using space-time filtering, following the method of Kiladis (19). This approach involves an inverse Fourier transform in the longitude and time dimensions, setting all spectral coefficients outside the targeted ranges to zero. For the MJO frequency, we select eastward wavenumbers between 1 and 8 and a wave period range of 30 to 96 d.

The MJO composite analysis is performed by averaging different variables during active and inactive MJO periods. We first identify the peak day of ${\bar{T}}_{\text{500,f}}$, defined as the day with the maximum value within a 20-d window. An active MJO event is defined as a case where the RMM amplitude, smoothed with a 10-d running mean, remains above 1 throughout the 20-d window. Conversely, an inactive MJO event is one in which the smoothed RMM amplitude remains below 1 for the entire window. Applying these criteria yields a total of 55 active MJO cases and 27 inactive cases. We assess the robustness of the spatial patterns in the active and inactive MJO composites using a Student’s *t* test.

The degree of mesoscale organization is quantified using a clustering index, $I_{\text{org}}$ (54). This nondimensional metric assesses convective organization by comparing the nearest-neighbor distance distribution of convective centroids to that of a random distribution with the same number of clusters. To compute $I_{\text{org}}$, we use 20 y of daily precipitation data from the Integrated Multi-satellite Retrievals for GPM IMERG (55). Following Bao et al. (56), we first identify convective regions as grid cells where daily precipitation exceeds the 95th quantile over the entire tropical domain (30°N–30°S) on a given day. Convective clusters are then defined by connected grid cells, and cluster centroids are located at the local maxima of daily precipitation within each cluster. The resulting $I_{\text{org}}$ values range from 0 to 1, with higher values indicating greater convective organization.

## Supplementary Material

Appendix 01 (PDF)

## Data Availability

Code and scripts have been deposited in Zenodo (DOI: 10.5281/zenodo.15366504) (57). All other data are included in the manuscript and/or *SI Appendix*.

## References

[1] H. Riehl, J. S. Malkus, On the heat balance in the equatorial trough zone. Geophysica **6**, 503–538 (1958).

[2] J. Bjerknes, Atmospheric teleconnections from the equatorial pacific. Mon. Weather. Rev. **97**, 163–172 (1969).

[3] T. Matsuno, Quasi-geostrophic motions in the equatorial area. J. Meteorol. Soc. Jpn. Ser. II **44**, 25–43 (1966).

[4] R. A. Madden, P. R. Julian, Detection of a 40–50 day oscillation in the zonal wind in the tropical pacific. J. Atmos. Sci. **28**, 702–708 (1971).

[5] C. Zhang, Madden-Julian Oscillation: Bridging weather and climate. Bull. Am. Meteorol. Soc. **94**, 1849–1870 (2013).

[6] J. Dias, N. Sakaeda, G. N. Kiladis, K. Kikuchi, Influences of the MJO on the space-time organization of tropical convection. J. Geophys. Res. Atmos. **122**, 8012–8032 (2017).

[7] M. Wheeler, G. N. Kiladis, Convectively coupled equatorial waves: Analysis of clouds and temperature in the wavenumber-frequency domain. J. Atmos. Sci. **56**, 374–399 (1999).

[8] S. Bony , Observed modulation of the tropical radiation budget by deep convective organization and lower-tropospheric stability. AGU Adv. **1**, e2019AV000155 (2020).

[9] S. Manabe, R. F. Strickler, Thermal equilibrium of the atmosphere with a convective adjustment. J. Atmos. Sci. **21**, 361–385 (1964).

[10] Q. Hu, D. A. Randall, Low-frequency oscillations in radiative-convective systems. J. Atmos. Sci. **51**, 1089–1099 (1994).

[11] I. M. Held, R. S. Hemler, V. Ramaswamy, Radiative-convective equilibrium with explicit two-dimensional moist convection. J. Atmos. Sci. **50**, 3909–3927 (1993).

[12] A. M. Tompkins, Organization of tropical convection in low vertical wind shears: The role of water vapor. J. Atmos. Sci. **58**, 529–545 (2001).

[13] C. S. Bretherton, P. N. Blossey, M. Khairoutdinov, An energy-balance analysis of deep convective self-aggregation above uniform SST. J. Atmos. Sci. **62**, 4273–4292 (2005).

[14] C. J. Muller, I. M. Held, Detailed investigation of the self-aggregation of convection in cloud-resolving simulations. J. Atmos. Sci. **69**, 2551–2565 (2012).

[15] K. Emanuel, A. A. Wing, E. M. Vincent, Radiative-convective instability. J. Adv. Model. Earth Syst. **6**, 75–90 (2014).

[16] D. Coppin, S. Bony, Internal variability in a coupled general circulation model in radiative-convective equilibrium. Geophys. Res. Lett. **44**, 5142–5149 (2017).

[17] A. B. Sokol, V. A. Munteanu, P. N. Blossey, D. L. Hartmann, Internal ocean-atmosphere variability in kilometer-scale radiative-convective equilibrium. J. Adv. Model. Earth Syst. **17**, e2024MS004567 (2025).

[18] P. E. Roundy, W. M. Frank, A climatology of waves in the equatorial region. J. Atmos. Sci. **61**, 2105–2132 (2004).

[19] G. N. Kiladis, M. C. Wheeler, P. T. Haertel, K. H. Straub, P. E. Roundy, Convectively coupled equatorial waves. Rev. Geophys. **47**, e2008RG000266 (2009).

[20] A. E. Gill, Some simple solutions for heat-induced tropical circulation. Q. J. R. Meteorol. Soc. **106**, 447–462 (1980).

[21] Y. N. Takayabu, M. Murakami, The structure of super cloud clusters observed in 1–20 June 1986 and their relationship to easterly waves. J. Meteorol. Soc. Jpn. Ser. II **69**, 105–125 (1991).

[22] C. Zhang, A. F. Adames, B. Khouider, B. Wang, D. Yang, Four theories of the Madden-Julian Oscillation. Rev. Geophys. **58**, e2019RG000685 (2020). 10.1029/2019RG000685 PMC7375192 32879923

[23] D. Yang, Á. F. Adames, B. Khouider, B. Wang, C. Zhang, “A review of contemporary MJO theories” in World Scientific Series on Asia-Pacific Weather and Climate: The Multiscale Global Monsoon System, C.-P. Chang , Eds. (World Scientific, 2021), vol. 11, chap. 19, pp. 233–247.

[24] S. Bony , Clouds, circulation and climate sensitivity. Nat. Geosci. **8**, 261–268 (2015).

[25] N. P. Arnold, D. A. Randall, Global-scale convective aggregation: Implications for the Madden-Julian Oscillation. J. Adv. Model. Earth Syst. **7**, 1499–1518 (2015).

[26] M. F. Khairoutdinov, K. Emanuel, Intraseasonal variability in a cloud-permitting near-global equatorial aquaplanet model. J. Atmos. Sci. **75**, 4337–4355 (2018).

[27] S. Bony , Robust direct effect of carbon dioxide on tropical circulation and regional precipitation. Nat. Geosci. **6**, 447–451 (2013).

[28] C. S. Bretherton, P. K. Smolarkiewicz, Gravity waves, compensating subsidence and detrainment around cumulus clouds. J. Atmos. Sci. **46**, 740–759 (1989).

[29] A. H. Sobel, C. S. Bretherton, Modeling tropical precipitation in a single column. J. Clim. **13**, 4378–4392 (2000).

[30] M. C. Wheeler, H. H. Hendon, An all-season real-time multivariate MJO index: Development of an index for monitoring and prediction. Mon. Weather. Rev. **132**, 1917–1932 (2004).

[31] K. H. Straub, MJO initiation in the real-time multivariate MJO index. J. Clim. **26**, 1130–1151 (2013).

[32] G. N. Kiladis , A comparison of OLR and circulation-based indices for tracking the MJO. Mon. Weather. Rev. **142**, 1697–1715 (2014).

[33] O. Bühler, WAVE-MEAN INTERACTION THEORY, Cambridge Monographs on Mechanics (Cambridge University Press, 2014), pp. 99–100.

[34] A. F. Adames, J. M. Wallace, Three-dimensional structure and evolution of the vertical velocity and divergence fields in the MJO. J. Atmos. Sci. **71**, 4661–4681 (2014).

[35] K. S. Virts, J. M. Wallace, Observations of temperature, wind, cirrus, and trace gases in the tropical tropopause transition layer during the MJO. J. Atmos. Sci. **71**, 1143–1157 (2014).

[36] A. I. L. Williams, N. Jeevanjee, J. Bloch-Johnson, Circus tents, convective thresholds, and the non-linear climate response to tropical SSTs. Geophys. Res. Lett. **50**, e2022GL101499 (2023).

[37] A. O. Gonzalez, X. Jiang, Distinct propagation characteristics of intraseasonal variability over the tropical west pacific. J. Geophys. Res. Atmos. **124**, 5332–5351 (2019).

[38] V. C. Mayta, A. F. Adames, F. Ahmed, Westward-propagating moisture mode over the tropical western hemisphere. Geophys. Res. Lett. **49**, e2022GL097799 (2022).

[39] A. A. Wing, L. G. Silvers, K. A. Reed, Rcemip-II: Mock-walker simulations as phase II of the radiative-convective equilibrium model intercomparison project. Geosci. Model. Dev. **17**, 6195–6225 (2024).

[40] K. Emanuel, Inferences from simple models of slow, convectively coupled processes. J. Atmos. Sci. **76**, 195–208 (2019).

[41] K. A. Emanuel, J. David Neelin, C. S. Bretherton, On large-scale circulations in convecting atmospheres. Q. J. R. Meteorol. Soc. **120**, 1111–1143 (1994).

[42] C. Jakob, M. S. Singh, L. Jungandreas, Radiative convective equilibrium and organized convection: An observational perspective. J. Geophys. Res. Atmos. **124**, 5418–5430 (2019).

[43] K. E. Trenberth, J. M. Caron, Estimates of meridional atmosphere and ocean heat transports. J. Clim. **14**, 3433–3443 (2001).

[44] E. N. Lorenz, The nature and theory of the general circulation of the atmosphere. Bull. Am. Meteorol. Soc. **218**, 161 (1967).

[45] I. M. Held, A. Y. Hou, Nonlinear axially symmetric circulations in a nearly inviscid atmosphere. J. Atmos. Sci. **37**, 515–533 (1980).

[46] K. M. Weickmann, P. D. Sardeshmukh, The atmospheric angular momentum cycle associated with a Madden-Julian Oscillation. J. Atmos. Sci. **51**, 3194–3208 (1994).

[47] K. Weickmann, E. Berry, A synoptic-dynamic model of subseasonal atmospheric variability. Mon. Weather. Rev. **135**, 449–474 (2007).

[48] K. Weickmann, E. Berry, The tropical Madden-Julian Oscillation and the global wind oscillation. Mon. Weather. Rev. **137**, 1601–1614 (2009).

[49] H. Hersbach , The ERA5 global reanalysis. Q. J. R. Meteorol. Soc. **146**, 1999–2049 (2020).

[50] N. G. Loeb , Clouds and the Earth’s Radiant Energy System (CERES) Energy Balanced and Filled (EBAF) Top-of-Atmosphere (TOA) Edition-4.0 data product. J. Clim. **31**, 895–918 (2018).

[51] B. Huang , Improvements of the daily optimum interpolation sea surface temperature (DOISST) version 2.1. J. Clim. **34**, 2923–2939 (2021).

[52] R. Gelaro , The modern-era retrospective analysis for research and applications, version 2 (MERRA-2). J. Clim. **30**, 5419–5454 (2017). 10.1175/JCLI-D-16-0758.1 PMC6999672 32020988

[53] R. E. Livezey, W. Y. Chen, Statistical field significance and its determination by Monte Carlo techniques. Mon. Weather. Rev. **111**, 46–59 (1983).

[54] A. M. Tompkins, A. G. Semie, Organization of tropical convection in low vertical wind shears: Role of updraft entrainment. J. Adv. Model. Earth Syst. **9**, 1046–1068 (2017).

[55] G. Huffman, E. Stocker, D. Bolvin, E. Nelkin, T. Jackson, GPM IMERG Final Precipitation L3 Half Hourly 0.1 degree x 0.1 degree V06 (Greenbelt, MD, Goddard Earth Sciences Data and Information Services Center (GES DISC), 2019). Accessed 11 May 2021.

[56] J. Bao, B. Stevens, L. Kluft, C. Muller, Intensification of daily tropical precipitation extremes from more organized convection. Sci. Adv. **10**, eadj6801 (2024). 38394192 10.1126/sciadv.adj6801 PMC10889435

[57] Bao , Codes and scripts for “Tropics-wide intraseasonal oscillation.” Zenodo. 10.5281/zenodo.15366504. Deposited 15 October 2025.

