# Supplementary material for: Tropics-wide intraseasonal oscillations
Source: Proc Natl Acad Sci U S A. 2025 Nov 24;122(48):e2511549122. doi: 10.1073/pnas.2511549122 (PMC12685094; doi:10.1073/pnas.2511549122)
Supplement: Supplementary file 1 — Appendix 01 (PDF) [file pnas.2511549122.sapp.pdf]

PNAS

Supporting Information for

Tropics-wide intraseasonal oscillation

Jiawei Bao, Sandrine Bony, Daisuke Takasuka and Caroline Muller

Jiawei Bao
E-mail: jiawei.bao@ist.ac.at

This PDF file includes:

Figs. S1 to S8

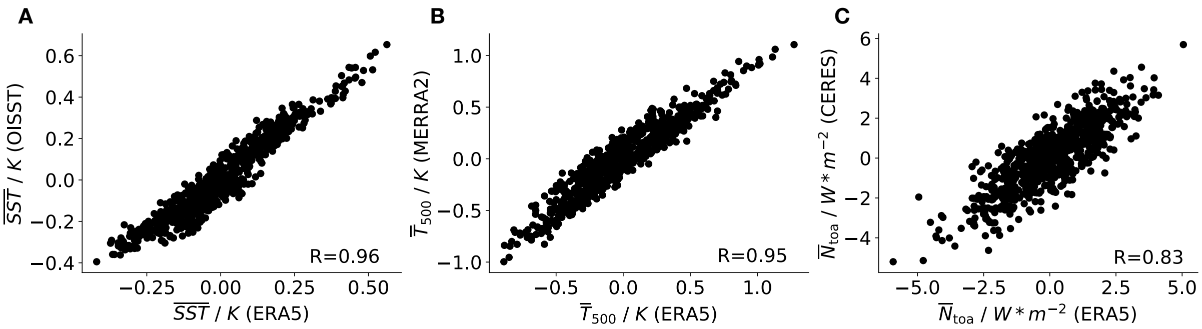

Fig. S1. Scatter plots of tropical-mean daily sea surface temperature ($\overline{SST}$, A), atmospheric temperature at 500hPa ($\overline{T}_{500}$, B) and net top-of-atmosphere atmopsheric radiation ($\overline{N}_{\mathrm{toa}}$, C) averaged over the whole tropics (30°S to 30°N) from ERA5 vs other data products including $\overline{SST}$ data from OISST, $\overline{T}_{500}$ from MERRA2 and $\overline{N}_{\mathrm{toa}}$ from CERES. The data are deseasonalized by subtracting the 20-year (2001-2020) daily climatological values.

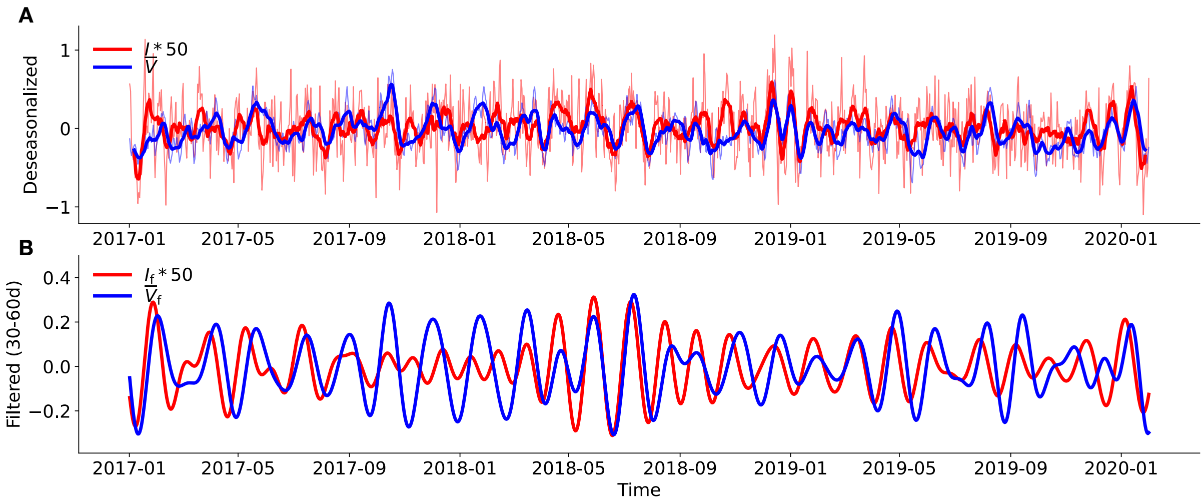

Fig. S2. (A): Time series of large-scale pressure vertical velocity difference ($I = \overline{\omega}_{dn} - \overline{\omega}_{up}$) and tropical mean surface wind speed ($\overline{V}$). I is calculated with data between 20°S to 20°N while $\overline{V}$ is calculated with data between 30°S to 30°N. The data are deseasonalized by subtracting the 20-year daily climatological values. Thin lines: the original time series after deseasonalization; thick lines: the smoothed time series obtained by applying a 10-day running mean; (B): The same timeseries as in (A) but filtered over 30-60 days. Note that the time series of pressure vertical velocity difference are scaled by a factor of 50 to facilitate comparison.

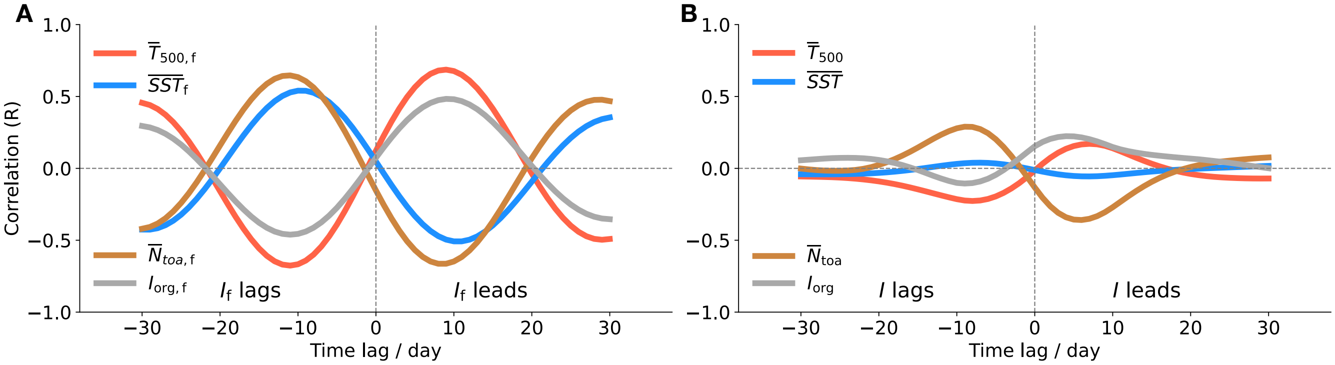

Fig. S3. (A) Lead-lag correlation coefficients between daily time series of filtered tropical mean overturning circulation index: I_{f} and other variables (including 500hPa temperature: $\overline{T}_{500,\mathrm{f}}$, sea surface temperature: $\overline{SST}_{\mathrm{f}}$, top-of-atmosphere radiation: $\overline{N}_{\mathrm{toa,f}}$ and convective organization: $I_{\mathrm{org,f}}$) filtered over 30-60 days. (B) Lead-lag correlation coefficients between daily time series (10-day running mean) of the deseasonalized tropical mean overturning circulation index: I and other variables (including 500hPa temperature: $\overline{T}_{500}$, sea surface temperature: $\overline{SST}$, top-of-atmosphere radiation: $\overline{N}_{\mathrm{toa}}$ and convective organization: I_{org}).

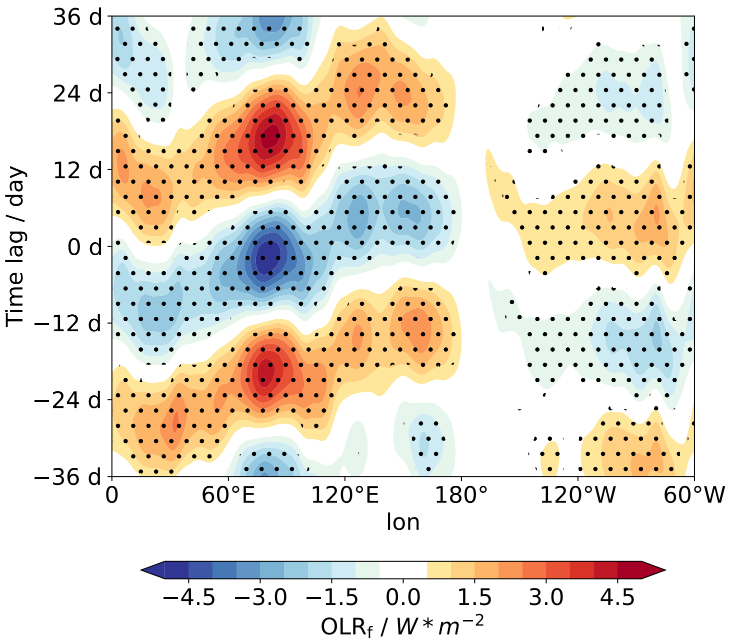

Fig. S4. Hovmöller diagram of composite OLR$_\mathrm{f}$ associated with peaks in the tropical mean overturning circulation index I_f. OLR is averaged meridionally between 10°S and 10°N and filtered in the 30–60-day band. Peak I_f days are defined as those on which I_f exceeds its values during both the 20 days before and the 20 days after. Regions where results are significant at the 95 % confidence level (p $<$ 0.05) are indicated by stippling.

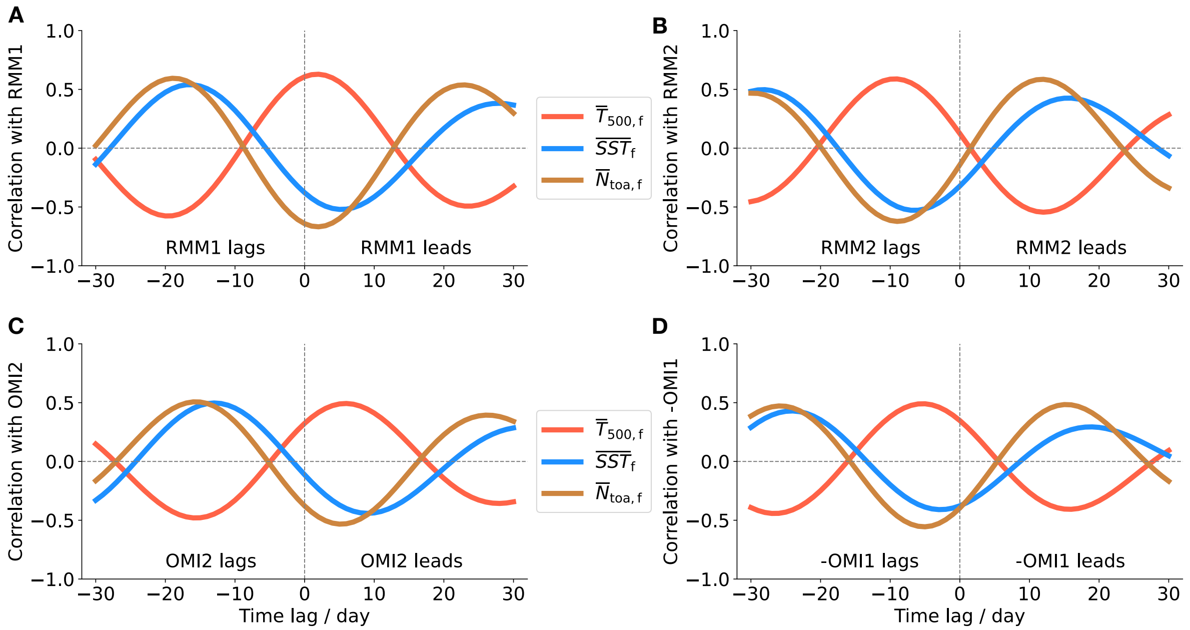

Fig. S5. Lead-lag correlation coefficient between (A) RMM1 index, (B) RMM2 index, (C) OMI2 index, (D) -OMI1 index with daily time series of 500hPa temperature: $\overline{T}_{500,\mathrm{f}}$, sea surface temperature: $\overline{SST}_\mathrm{f}$ and top-of-atmosphere radiation: $\overline{N}_{\mathrm{toa,f}}$ filtered over 30-60 days.

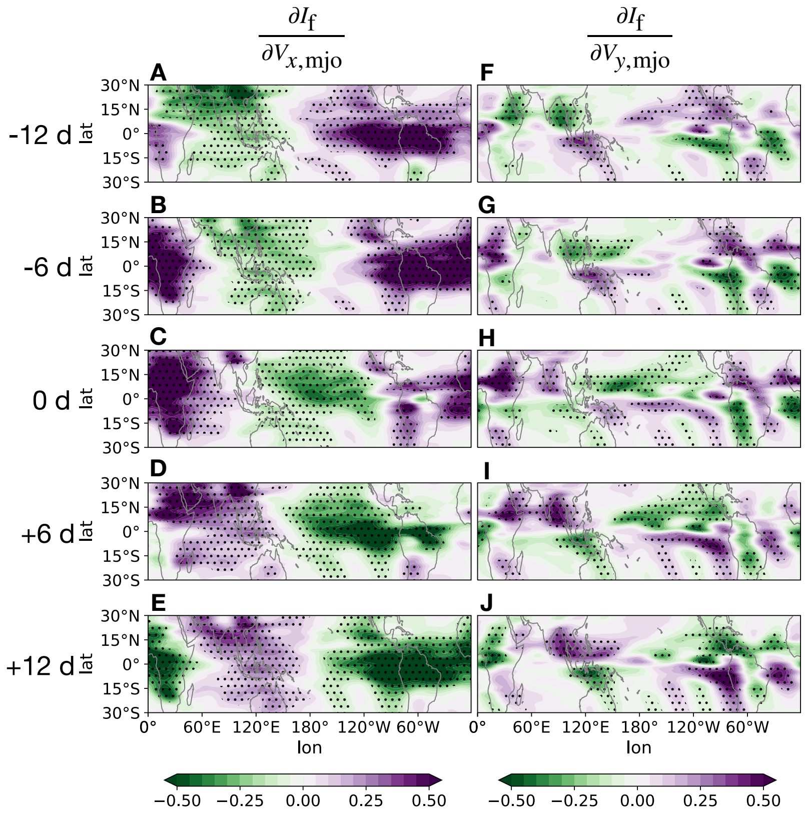

Fig. S6. Spatial map of lead-lag regression coefficients by regressing the time series of filtered tropical mean overturning circulation index $I_{\rm f}$ onto the time series of MJO-filtered surface zonal wind $V_{x,\rm mjo}$ (A-E) and meridional wind $V_{y,\rm mjo}$ (F-J) at each grid point across the entire tropical domain. All variables are filtered over 30-60 days. From top to bottom shows 12-day lag, 6-day lag, no lag, 6-day lead and 12-day lead of $I_{\rm f}$. Regression coefficients are normalized by the ratio of the standard deviations of the two variables, calculated across all spatial and temporal dimensions, to make them unitless. Regions where results are significant at the 95 % confidence level ($p < 0.05$) are indicated by stippling.

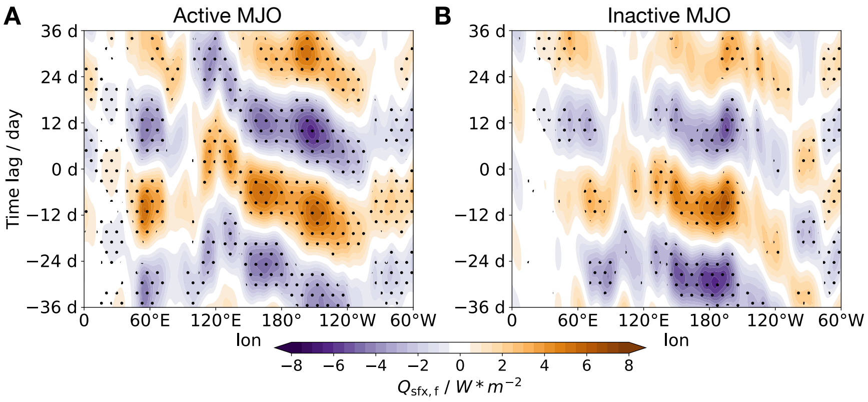

Fig. S7. Hovmöller diagrams of composite filtered surface heat fluxes ($Q_{\mathrm{sfx,f}}$) associated with $\overline{T}_{500,\mathrm{f}}$ peaks at day 0 during active MJO (A) and inactive MJO (B) periods. $Q_{\mathrm{sfx,f}}$ is averaged meridionally between 20°S and 20°N and filtered in the 30–60-day band. Stippling indicates regions where anomalies are statistically significant at the 95% confidence level ($p < 0.05$).

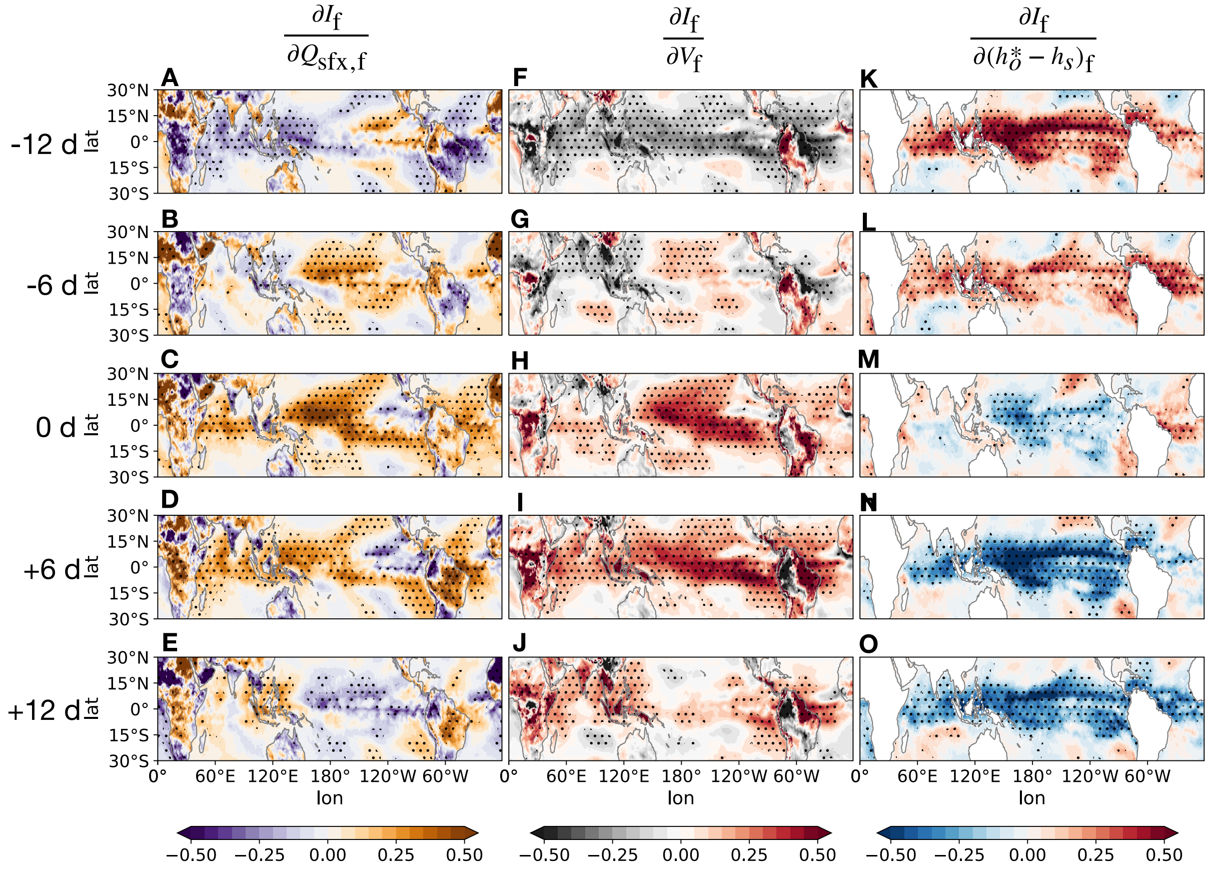

Fig. S8. Spatial map of lead-lag regression coefficients by regressing the time series of filtered tropical mean overturning circulation index I_f onto the time series of other variables, including surface fluxes $Q_{sfx,f}$ (A-E), surface wind speed V_f (F-J), and air-sea enthalpy differences $(h_o^* - h_s)_f$ (K-O) at each grid point across the entire tropical domain. All variables are filtered over 30-60 days. From top to bottom shows 12-day lag, 6-day lag, no lag, 6-day lead and 12-day lead of I_f. Regression coefficients are normalized by the ratio of the standard deviations of the two variables, calculated across all spatial and temporal dimensions, to make them unitless. Regions where results are significant at the 95 % confidence level ($p < 0.05$) are indicated by stippling.
